# Frozen Emotions, Frozen Bodies: Limbic Involvement in Catatonia. A Systematic Review

**DOI:** 10.1192/j.eurpsy.2026.10923

**Published:** 2026-07-31

**Authors:** F. Marcolini, D. Tartarini, V. Beghelli, V. Di Giacomo, D. De Ronchi, A. R. Atti

**Affiliations:** 1 Department of Biomedical and Neuromotor Sciences, University of Bologna, Bologna; 2 Department of Mental and Physical Health and Preventive Medicine, Università degli Studi della Campania “Luigi Vanvitelli”, Naples, Italy

## Abstract

**Introduction:**

Catatonia is a multifaceted neuropsychiatric condition characterized by both motor and affective disturbances. While existing neurobiological frameworks have largely emphasized cortical and cerebellar dysfunctions to explain its sensorimotor and behavioral features, the contribution of the limbic system remains less investigated. Given its central role in emotional regulation and motivational processes, emerging evidence suggests that limbic alterations may represent a crucial mechanism underlying catatonia, potentially offering a more comprehensive explanatory model and guiding therapeutic innovation.

**Objectives:**

This review aims to synthesize current knowledge on structural and functional abnormalities of the limbic system in catatonia. Specific objectives include mapping the implicated limbic regions, clarifying their possible role in symptom expression, and considering how these insights may inform targeted clinical interventions.

**Methods:**

The review was carried out according to PRISMA guidelines. Systematic searches were performed in MEDLINE, Web of Science, PsycINFO, and Scopus without temporal restrictions. We included studies involving patients with a standardized diagnosis of catatonia who underwent biological or instrumental investigations of limbic regions. Extracted data encompassed study design, sample characteristics, diagnostic framework, type of investigation, and limbic findings at the structural or functional level. Methodological quality was appraised using Joanna Briggs Institute tools, with disagreements resolved by discussion or external consultation.

**Results:**

The search strategy yielded 1792 records; after screening, 54 full texts were reviewed and 14 fulfilled eligibility criteria. Hand-searching of references added 5 further studies, resulting in 19 included articles: 7 case reports and 12 observational investigations, most of which applied neuroimaging to assess limbic involvement in catatonia. The anterior cingulate cortex emerged as the most consistently implicated area, with findings of altered gyrification, reduced grey matter, and perfusion changes associated with clinical manifestations. The orbitofrontal cortex - though not strictly part of the limbic system but functionally related - was also frequently reported as altered. Evidence concerning the amygdala, hypothalamus, and other structures was more heterogeneous, ranging from volumetric abnormalities to biochemical dysregulation linked to genetic or acquired factors.

**Conclusions:**

The review supports a significant contribution of limbic dysfunction to catatonia, adding to prevailing models centered on cortical and cerebellar pathology. Nevertheless, heterogeneity in diagnostic criteria, methodological limitations, and small sample sizes constrain the strength of current evidence. Future research with more rigorous designs is essential to clarify the role of limbic networks in catatonia and to advance treatment perspectives.

**Disclosure of Interest:**

None Declared

